# Injectable drug-loaded polysaccharide hybrid hydrogels for hemostasis

**DOI:** 10.1039/c9ra07116d

**Published:** 2019-11-12

**Authors:** Jinying Cao, Ling Xiao, Xiaowen Shi

**Affiliations:** School of Resource and Environmental Science, Key Laboratory for Biomass Resource Chemistry and Environmental Biotechnology of Hubei Province, Wuhan University Wuhan 430079 China 2013301110176@whu.edu.cn xiaoling9119@whu.edu.cn shixwwhu@163.com

## Abstract

An injectable hydrogel with high adhesion strength, non-toxicity and low cost is highly desired for developing highly efficient hemostasis. In this study, we developed a new type of injectable adhesive drug loaded hydrogel utilizing the formation of Schiff-base linkages based on carboxymethyl chitosan (CMC), gelatin (GEL) and oxidized alginate (OSA). By optimizing the concentration of the biopolymers, the hybrid hydrogel (CMC-GEL/OSA) demonstrated an extremely fast gelation rate (30 s) and adhesive strength of 11 kPa. The freeze-dried hydrogel showed a three-dimensional porous structure. The hydrogel loaded with levofloxacin exhibited good antibacterial properties. Hemostatic performance of the hydrogel was demonstrated in a rat liver injury model. Compared with the untreated wound, the hemostasis time of the hydrogel treated wound was shortened by 84.2% and the blood loss was reduced by 82.2%. Thus, the proposed injectable hydrogel holds great potential applications for hemostasis, drug delivery and in other biomedical fields.

## Abstract 2

A levofloxacin loaded CMC-GEL/OSA hydrogel was synthesized that exhibited good antibacterial properties, 84.2% shortened hemostatic time and 82.2% reduced blood loss.

## Graphical abstract

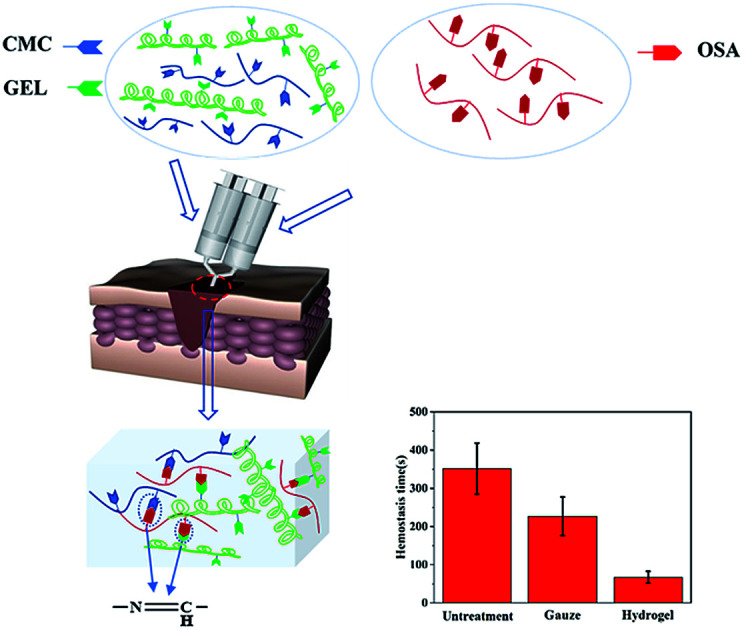

## 1. Introduction

Uncontrollable bleeding is the main cause of death from injuries that occur in battlefield, emergency and hospital settings. It has been reported that 50% of military deaths are the result of excessive bleeding. Thus, it is imperative to reduce unexpected blood loss in the prehospital setting to increase the survival rate of injuries. In the past decades, a variety of hemostatic technologies including glues, bandages, tourniquets, dressings, procoagulant powders, and injectable hydrogels and cryogels have been developed,^1–3^ among which, tissue adhesives have attracted much attention owing to their effective performance for wound closure and hemostasis. To some extent, tissue adhesives have revolutionized surgical procedures due to their substitution for pressing and suturing.^4^

An ideal tissue adhesive for hemostasis not only requires rapid adhesion to close wounds, but should also have low toxicity, good mechanical properties, be easy to use, and inexpensive.^5^ There are many tissue adhesives available, and each has its own advantages and disadvantages. Cyanoacrylate adhesives are often used medically because of their strong bonding strength and rapid curing rate in wet environments, but their degradation products are toxic and the curing process is exothermic, which limited their application.^6^ Fibrin glue, as a representative of biological agents, has been popularly used for many surgical procedures due to the characteristics of high hemostasis efficiency and no dependence on coagulation factors.^7^ However, the fibrin glue has some shortcomings, for example, poor tissue adhesion, great chance of contaminations with viruses and high cost.^8^ In addition, ethylene glycol based adhesives swell too much and are prone to compressing tissues in closed cavities,^4^ while some photopolymerizable adhesives require UV illumination and become inconvenient to use.^9,10^ Therefore, novel tissue adhesive with strong adhesion strength, suitable mechanical property, good biocompatibility and low cost is highly desirable.

Oxidized polysaccharides have gained much attention as a new type of cross-linking agents owing to low toxicity, good biocompatibility and biodegradability, among which oxidized sodium alginate (OSA) has outstanding performance.^11–15^ Sodium alginate composed of 1,4-linked β-d-mannuronate (M) and 1,4-linked α-l-guluronate (G) is a variety of anionic linear polysaccharides derived from algae and bacteria, and has been widely used in biomedical field due to the biocompatibility and low cost.^14^ It has been widely studied that sodium alginate is oxidized to transform adjacent hydroxyl groups into aldehyde groups and reacts with amino containing polymers to form *in situ* hydrogels *via* Schiff base reaction.^14^ Oxidized alginate can rapidly cross-link gelatin in the presence of borax to form hydrogels that are both non-toxic and biodegradable, which is a classic reaction and has potential application in wound pressing, cell encapsulation and cartilage regeneration.^16–18^ With oxidized alginate as cross-linking agent, carboxymethyl chitosan (CMC) based and glycol chitosan based hydrogels have been formed respectively and used in drug delivery and tissue engineering.^19,20^ However, the hydrogels formed by simple cross-linking between oxidized alginate and gelatin or carboxymethyl chitosan may not have enough gel strength and adhesive strength, which impedes their application in hemostasis.^1,19^ To overcome such challenges, some efforts have been made. Wu *et al.* and Yuan *et al.* respectively connected hexamethylene diamine to gelatin and carboxymethyl chitosan to increase the amino content, resulting in less gelling time and higher adhesion strength.^21,22^ Wei *et al.* developed a novel double cross-linking hydrogel utilizing the dynamic reaction of *N*-carboxyethyl chitosan and adipic acid dihydrazide with oxidized sodium alginate, and a high healing efficiency (up to 95%) was obtained.^23^ In order to increase the amino group content to promote adhesion strength, and construct double-network to increase mechanical strength, we blended gelatin with carboxymethyl chitosan and cross-linked them with oxidized alginate to prepare injectable tissue adhesive.

In this study, injectable hydrogels were prepared through the *in situ* formation of Schiff-base linkages between gelatin, carboxymethyl chitosan, and oxidized sodium alginate. In order to ensure the injectable property of the gel at room temperature, the gelatin from cold-water fish skin, whose melting point is 6 °C,^24^ was used. The physicochemical properties, including gelation time, lap shear strength and rheological analysis of the carboxymethyl chitosan-gelatin/oxidized sodium alginate (CMC-GEL/OSA) hybrid hydrogels were characterized. In order to suppress possible infection of the wound, levofloxacin was loaded in the hybrid hydrogel and the release behavior of levofloxacin was studied. Further, the inhibition zone test demonstrated the enhancement of the anti-bacterial property of the hydrogel. Finally, the hemostatic efficacy of the hydrogel was estimated in a rat liver injury model.

## 2. Materials and methods

### 2.1. Materials

Cold-water fish skin ‘‘type A” gelatin (G7041) was purchased from Sigma-Aldrich (Shanghai, China). *N*,*O*-Carboxymethyl chitosan (substitution degree of ≥80%, deacetylation degree of 90%) was provided by Dubai Biotechnology Co. Ltd. (Shanghai, China). Sodium alginate (viscosity of ≥200 mPa s), sodium periodate and levofloxacin were purchased from Sinopharm Chemical Reagent (Beijing, China). All other reagents were of analytical grade and used without further purification.

### 2.2. Preparation of oxidized sodium alginate

Sodium alginate with 50% degree of oxidation was prepared according to previous literatures.^25,26^ A 20 g portion of sodium alginate was dispersed in 100 mL ethanol, and then 100 mL of ultrapure water containing 10.9 g sodium periodate was added into the above solution. The mixture was magnetically stirred in the dark at 25 °C for 6 h. The reaction was terminated by adding ethylene glycol (5 mL) and stirring for additional 1 h. The crude product was obtained by adding 1.0 L ethanol and suction filtration. Then, the product was dialyzed (MWCO = 3.5 kDa) for 3 days and lyophilized.

### 2.3. Preparation of hydrogels and gelling time measurement

Oxidized sodium alginate solutions (15 wt% in PBS buffer solution, pH 7.4) were mixed with equal volume of aqueous solution containing carboxymethyl chitosan and gelatin at different concentrations. The gelation time of various formulations was determined by an inverted tube test.^27^ Briefly, the solutions were mixed in a 5 mL centrifuge tube, and the gelation time was determined by inverting the tube every 5 s after the initial mixing process. All measurements were repeated in triplicates.

### 2.4. Lap shear test

The lap shear strength of the hydrogels formed by different formulations was assessed according to the standard test method ASTM F2255-05 using an Electromechanical Universal Testing Machine (CMT6503, MTS Systems, China). Briefly, the mixture of 20 μL carboxymethyl chitosan and gelatin solution and 20 μL oxidized sodium alginate solution was applied on the top region (10 mm × 25 mm) of one porcine skin sheet (45 mm × 25 mm), and the second porcine skin sheet was immediately set over this area. Afterwards, the smeared area of the two porcine skin sheets was pressed with a weight of 120 g. The specimen was placed in chamber at 25 °C for 1 h. The two porcine skin slides were placed into the machine for shear test by tensile loading with a strain rate of 1 mm min^−1^. The shear strength of the specimen was determined at the point of detaching. The measurements were repeated five times for each sample.

### 2.5. Characterization

The hydrogel was freeze-dried and the FT-IR spectrum was obtained from a FT-IR instrument (Nicolet 5700, Thermo, USA) by the KBr tabletting method. The hydrogel was freeze-dried and sprayed with gold, and the cross-section morphology of the composite gel was observed by SEM (EM-30 Plus, COXEM, Korea).

### 2.6. Rheological test

Rheological measurements were conducted using parallel plate geometry in a rotational rheometer (Kinexus pro+, Malvern Panalytical, UK) at a constant temperature of 25 °C. The hydrogel (15 mm diameter and 6 mm thickness) was placed on the lower plate 24 h after casting for strain and frequency sweep. The gap was set at 5.5 mm. The strain sweep was performed at a fixed frequency of 1 Hz under the linear viscoelastic range where the storage (*G*′) and loss (*G*′′) moduli of gels were independent of strain. The frequency sweep was performed by varying the frequency from 1 to 10 Hz when the strain was kept at 1%. For time sweep analysis, carboxymethyl chitosan and gelatin solution and oxidized sodium alginate solution were rapidly mixed on the lower parallel plate of the rheometer, and the storage moduli (*G*′) and loss moduli (*G*′′) were monitored as a function of time at frequency 1 Hz and strain 1%.

### 2.7. Levofloxacin release study

The release behavior of levofloxacin from the hydrogel was evaluated. Briefly, 0.5 g carboxymethyl chitosan, 2 g gelatin, 2 mg levofloxacin and 10 mL ultrapure water were mixed to form a solution and cross-linked with oxidized sodium alginate solution to prepare gel. One hour later, the gel was then immersed in 5 mL PBS buffer solution (pH 7.4) and placed in a 37 °C incubator. At regular interval time (1, 2, 3, 4, 5, 6, 12 and 24 hours), 5 mL solution was collected and replaced by fresh PBS solution. The absorbance of levofloxacin was read at 294 nm in an ultraviolet-visible spectrophotometer. The concentration of levofloxacin was obtained according to the standard curve, and the cumulative release was then calculated.

### 2.8. Inhibition zone test

The anti-bacterial property of the hydrogels was tested by inhibition zone. Briefly, beef extract (0.5 g), tryptone (1 g), NaCl (0.5 g) and agar (2 g) were dissolved in 100 mL ultrapure water and heated to melt, and sterilized under high pressure for about 30 min at 121 °C. The suspensions of *Escherichia coli* and *Staphylococcus aureus* were added to the Petri dishes respectively. When the medium was cooled and solidified, the gel was cut into circle and attached to the corresponding area of culture dish. Afterwards, the plates were inverted and incubated at 37 °C for 24 h. After incubation, the bacterial inhibition halos around the hydrogel specimens were observed.

### 2.9. Cytotoxicity evaluation

Cytotoxicity of the hydrogels was measured by contacting hydrogel extracts with L929 mouse fibroblast monolayers according to ISO standards (ISO 10993). Firstly, the extracts were obtained by culturing the hydrogel samples in Modified Eagle's Medium (MEM) at an extraction ratio of 0.1 g mL^−1^ for 24 h at 37 °C. After incubation, the extract was sterilized by 0.22 μm filters and 10% fetal bovine serum (FBS) was added. MEM medium was used as negative control. Subsequently, L929 mouse fibroblast suspension (100 μL) was inoculated into 96-well culture plate with a density of 5 × 10^3^ cells per well. The plate was incubated at 37 °C in 5% CO_2_ for 24 h. After incubation, the culture media was removed and hydrogel extract was used instead. After another 24 h, 10 μL CCK-8 solution was added to each well and incubated for a further 1 h. Then, the product was placed in a Microplate Reader to measure the absorbance at 450 nm wavelength. Cell viability was calculated by the following formula:
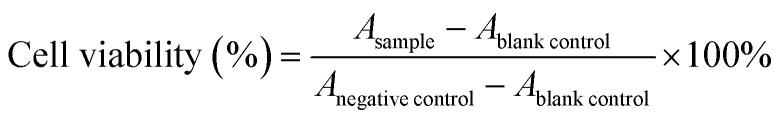


### 2.10. *In vivo* hemostatic test

In order to examine the efficacy of the hydrogel as tissue adhesive for liver injuries *in vivo*, a rat (normal SD rat, 185–200 g, 6–8 weeks, male) hemorrhaging liver model was developed according to previous literature.^28^ In brief, a rat was intraperitoneally injected with 35 mg kg^−1^ pentobarbital sodium and fixed on the operating table after anesthesia. After disinfection with iodine and alcohol cotton ball, the abdominal cavity was opened along the midline of the abdomen to expose the liver. In one lobe of the liver, a wound about 1 cm in length and 2 mm in depth was made with a scalpel and 300 μL of hydrogel was immediately injected to the surface of bleeding site. Hemostasis time and bleeding volume were observed and recorded. A total of three groups of experiments including blank control, gauze pressing and hydrogel were done, and each group contained five parallels. The animal procedures were performed in strict accordance with the Guidelines for Care and Use of Laboratory Animals of Wuhan University and the experiments were approved by the Animal Care and Use Committee of Wuhan University.

## 3 Results and discussion

### 3.1. Gelation of the composite gel

**Fig. 1:**
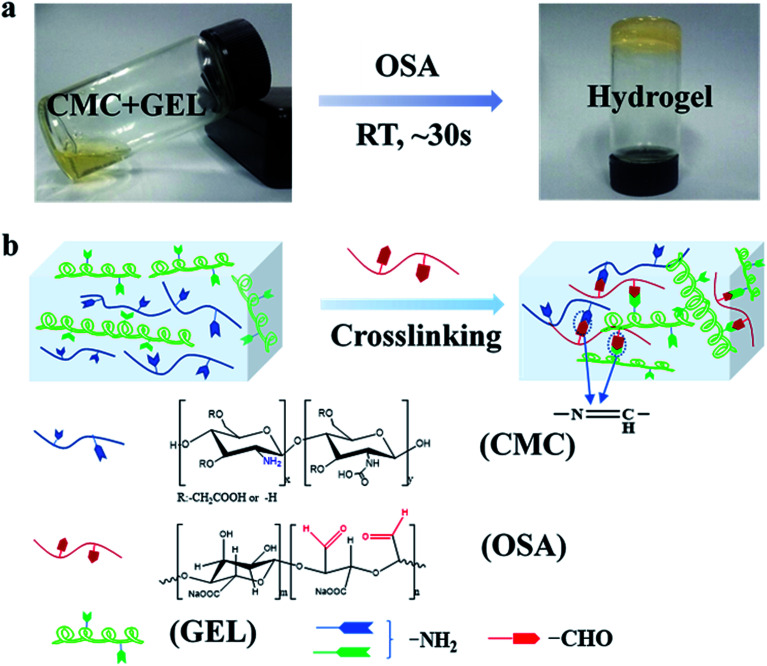
Schematic representation of the synthesis of CMC-GEL/OSA hydrogel. (a) Photographs of hydrogel formation; (b) chemical structure of the CMC-GEL/OSA hydrogel obtained by the reaction of the aldehyde groups (from OSA) and amino groups (from CMC and GEL).

The injectable hydrogel for hemostasis prefers to have a fast cross-linking to avoid hemostatic agent running away from the wound or being diluted. In this study, hydrogels were prepared by mixing equal volume of aqueous solution of carboxymethyl chitosan and gelatin with oxidized sodium alginate in PBS buffer solution. As shown in Fig. 1a, the mixture tuned to hydrogel at 30 s. The sol–gel transition of the hydrogel was caused by the *in situ* formation of Schiff base linkages between amino groups (from carboxymethyl chitosan and gelatin) and aldehyde groups (from oxidized sodium alginate) (Fig. 1b).

**Fig. 2:**
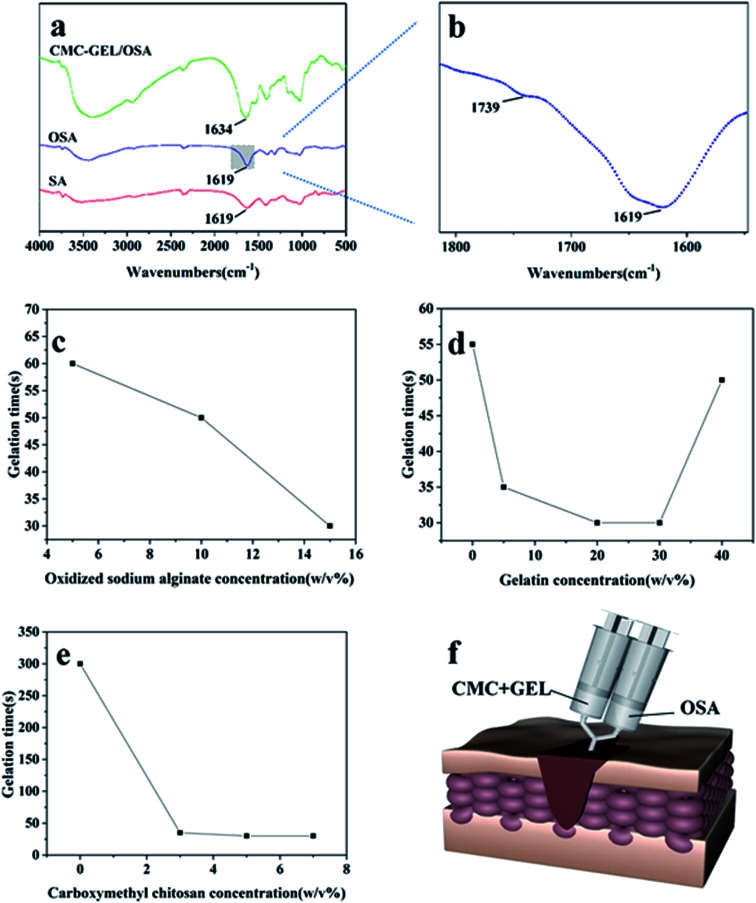
(a and b) Infrared spectra of SA, OSA and CMC-GEL/OSA hydrogel; (c) gelation time of hydrogels with different OSA concentration while the concentration of CMC and GEL was fixed at 5% and 20% respectively; (d) gelation time of hydrogels with different GEL concentration while the concentration of CMC and OSA was fixed at 5% and 15% respectively; (e) gelation time of hydrogels with different CMC concentration while the concentration of GEL and OSA was fixed at 20% and 15% respectively; (f) schematic diagram of gel injection.

Oxidized sodium alginate was synthesized by oxidation of sodium alginate with sodium periodate. Sodium alginate, oxidized sodium alginate and the CMC-GEL/OSA composite gel were dried and characterized by FT-IR, respectively (Fig. 2a). The locally enlarged FT-IR spectrum of oxidized sodium alginate was shown in Fig. 2b, and a characteristic peak at 1739 cm^−1^ corresponding to aldehyde group (–CHO) was observed, indicating the successful oxidation of C–OH.^29^ The peak was inconspicuous and this might be due to hemiacetal formation of free aldehyde groups.^29,30^ It has been reported that higher oxidation degree of sodium alginate increased the physiological toxicity and cross-linking effect.^31^ In order to balance both of them, the theoretical oxidation degree of 50% was selected for the preparation of oxidized sodium alginate in this study. The actual oxidation degree of oxidized sodium alginate measured by hydroxylamine hydrochloride titration was 50.2%, which was roughly consistent with the oxidation effect in previous literatures.^16,25,31^

The spectrum of the CMC-GEL/OSA hydrogel exhibited the characteristic absorption of imine stretching vibration (–C=N–) at 1634 cm^−1^ (Fig. 2a), indicating that dynamic Schiff bonds formed.^32^ Compared with sodium alginate and oxidized sodium alginate, the peaks of –OH at around 3400 cm^−1^ in the FT-IR spectrum of CMC-GEL/OSA had been widened and moved to a lower frequency, which indicated the presence of hydrogen bonds in the hydrogel.

### 3.2. Gelation time

The sol–gel transition of the hydrogel under different conditions is shown in Fig. 2c–e. The gelation time was evaluated by inverted tube test. It was shown that the oxidized sodium alginate concentration played an important role in gel formation. When 5% carboxymethyl chitosan and 20% gelatin solutions were mixed, there was no gel-forming observed. With the increased concentration of oxidized sodium alginate, the gel formed and the gelling time was obviously shortened (Fig. 2c). This could be attributed to the increase of aldehyde groups accelerated the synthetic rate of the Schiff base, as many literatures had been reported.^22,23,33^ However, when the oxidized sodium alginate concentration was too high, the solution became too viscous to inject. Thus, the concentration of oxidized sodium alginate was fixed at 15% in the subsequent experiments.

To further explore the gelation process, different formulations of carboxymethyl chitosan/gelatin solution were reacted with oxidized sodium alginate solution. Carboxymethyl chitosan and gelatin both played an important role in gelation process. When the concentration of gelatin was fixed at 20%, the gelation time was more than 5 minutes without carboxymethyl chitosan, and the gel time was shortened rapidly as carboxymethyl chitosan was added (Fig. 2d). This could be due to the hydrogen bond cross-linking between carboxymethyl chitosan and oxidized alginate as it had been found in FT-IR study. To confirm this, we added 15% oxidized sodium alginate into carboxymethyl chitosan solution whose pH was changed to 8.0 by NaOH, and the gelling time was ∼30 s, indicating the possible existence of hydrogen bond cross-linking between carboxymethyl chitosan and oxidized sodium alginate. When the concentration of carboxymethyl chitosan was fixed at 5%, with the concentration of gelatin increasing, the gel time firstly decreased and then increased (Fig. 2e). It could be due to that more amine groups accelerated the reaction of Schiff base. However, as the gelatin concentration was too high, there was no enough oxidized sodium alginate to react with amino groups, and the gel-forming process delayed. Above all, the hydrogel could be suitable for injection *via* the adjustment of component concentrations (Fig. 2f).

### 3.3. Evaluation of adhesion strength

**Fig. 3:**
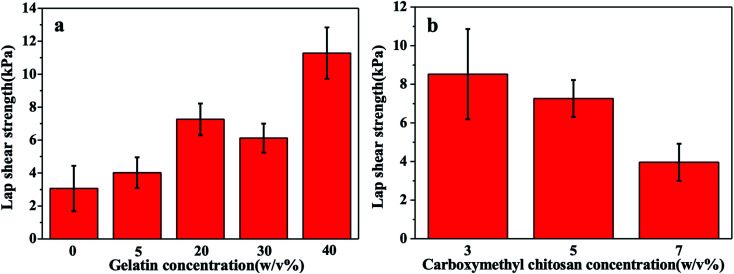
(a) Lap shear strength of hydrogels with different gelation concentration while the concentration of CMC and OSA was fixed at 5% and 15% respectively; (b) lap shear strength of hydrogels with different CMC concentration while the concentration of GEL and OSA was fixed at 20% and 15% respectively.

The adhesive strength is critical for the injectable hydrogel adhering to the wound. The adhesive strength of the gel is composed of cohesion and surface adhesion,^24^ and lap shear tensile test was carried out to illustrate the change of the gel adhesive strength with different formulations of the solution. As shown in Fig. 3a, the lap shear strength of the gel was improved with the increase of the concentration of gelatin, and the greatest adhesive strength reached 11 kPa, which was 3.7 folds of that without gelatin. This was owing to that gelatin provided more amino groups to enhance the reaction of Schiff base, and the double cross-linking network increased the gel strength. As shown in Fig. 3b, the lap shear strength of the gel decreased with the increase of the concentration of carboxymethyl chitosan, which might be attributed to the poorer cohesion strength caused by local over cross-linking.^34^

### 3.4. SEM

**Fig. 4:**
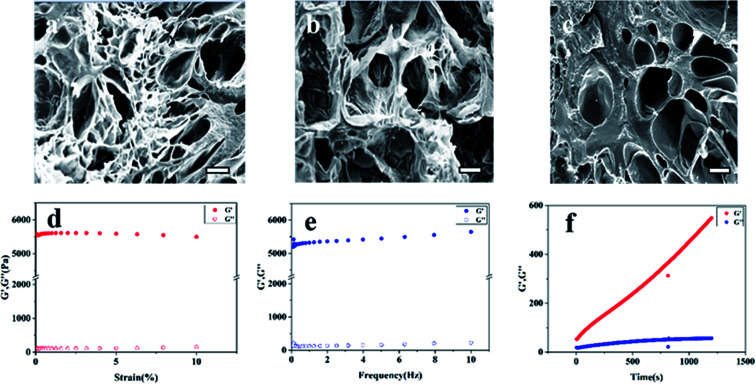
SEM diagram of the cross-section of the hydrogels (scale bar, 100 μm) (a) CMC/OSA, (b) GEL/OSA, (c) CMC-GEL/OSA. Rheological analysis of the CMC-GEL/OSA hydrogel (d) strain sweep (e) frequency sweep (f) time sweep. The concentration of CMC, GEL, and OSA was 5%, 20%, and 15%, respectively.

Considering the gelation time and adhesion strength, the compound gel of carboxymethyl chitosan 5% and gelatin 20% and oxidized sodium alginate 15% was chosen for further experiment. The SEM micrographs of the cross-section of hydrogels are presented in Fig. 4a–c. It was clearly observed that all the gels had three-dimensional porous structure, which was beneficial to the penetration of nutrients and the growth of tissue cells.^18^ In contrast to the GEL/OSA hydrogel, the pore distributions of CMC/OSA and CMC-GEL/OSA were not uniform, which might be due to local over cross-linking caused by the short reaction time.^34^

### 3.5. Rheological analysis

The rheological properties of the composite CMC-GEL/OSA hydrogel (5% CMC, 20% GEL, and 15% OSA) were measured by rotational rheometer. The results are shown in Fig. 4d–f. The linear viscoelastic range of the gel was determined by strain sweep, and it was seen that the modulus was independent of the strain up to 10% (Fig. 4d). Therefore, all further analyses were performed at 1% strain. The frequency sweep was performed by varying the frequency from 1 to 10 Hz. According to Fig. 4e, the gel strength almost had no dependence on the frequency, showing the characteristic of strong gel. Time sweep was done at strain 1% and frequency 1 Hz (Fig. 4f). The gelation was too rapid to record the gelling time at the crossing over point of the storage and loss modulus of the gel at the beginning of the test, but it was clear that the storage modulus (*G*′) of the gel was greater than the loss modulus (*G*′′), and the gel strength increased with time and reached about 550 Pa in 30 min, which indicated that the hydrogel was suitable for would hemostasis.

### 3.6. Release of levofloxacin and antibacterial property

**Fig. 5:**
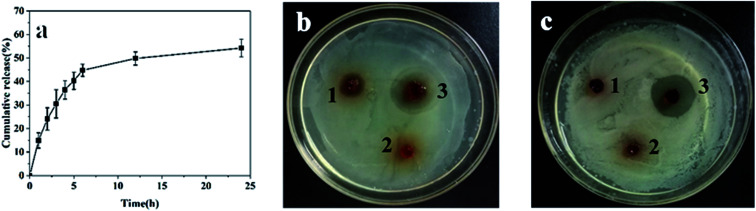
(a) Release curve of levofloxacin from the CMC-GEL/OSA hydrogel. Inhibition zone of (b) *Escherichia coli* and (c) *Staphylococcus aureus*, sample 1, 2 and 3 were CMC/OSA gel, CMC-GEL/OSA gel and levofloxacin loaded CMC-GEL/OSA gel, respectively. The concentrations of CMC, GEL, OSA and levofloxacin were 5%, 20%, 15%, and 200 mg kg^−1^, respectively.

For wound hemostasis, improving the antibacterial property can effectively prevent wound infection and is conducive to wound recovery. Levofloxacin is a synthetic antibiotic of the fluoroquinolone class, and has a broad-spectrum antibiotic effect on both Gram-positive and Gram-negative bacteria.^35^ In order to improve the antibacterial property and avoid infection, levofloxacin was loaded in the hydrogel. The release of levofloxacin was studied by immersing the gel in PBS solution (pH 7.4). As shown in Fig. 5a, the release rate of levofloxacin was fast within 0–6 h with 45% cumulative release, and then slowed down in the following 6–24 h with total 54% cumulative release at 24 h. Some levofloxacin retained in hydrogel, which might be due to the interaction between the residue amino and levofloxacin. Owing to the slow release and reservation of levofloxacin, the hydrogel was considered suitable to provide long-lasting bacteriostatic effect.

The antibacterial property of the gels was demonstrated by inhibition zone test. *Staphylococcus aureus* and *Escherichia coli* were selected as the representatives of Gram-positive and Gram-negative bacteria. As shown in Fig. 5, the CMC/OSA gel had a certain bacteriostatic effect on *Escherichia coli* due to the cationic group of carboxymethyl chitosan, while the bacteriostatic effect on *Staphylococcus aureus* was too weak to produce obvious inhibition.^36^ The CMC-GEL/OSA had no anti-bacterial properties which might be caused by the addition of GEL. Compared with the two kinds of gel mentioned above, the levofloxacin loaded gel produced obvious inhibition zone on both culture medium inoculated against *Staphylococcus aureus* and *Escherichia coli*. The strong antibacterial ability of CMC-GEL/OSA is beneficial for wound recovery.

### 3.7. Cytotoxicity evaluation and hemostatic property of CMC-GEL/OSA hydrogel

**Fig. 6:**
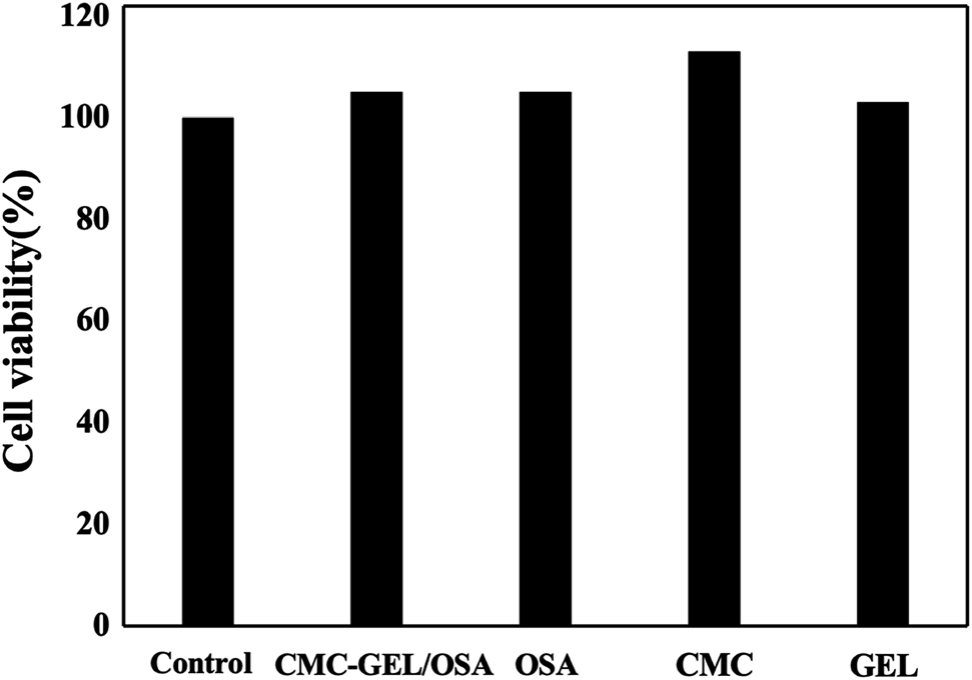
Cell viability of CMC-GEL/OSA, OSA, CMC and GEL.

The cell cytotoxicity was evaluated by incubating the hydrogel with L929 mouse fibroblast cells. The results in Fig. 6 showed that the hydrogel had no cytotoxicity to the cells due to the good viability of the cells (>100%). It suggests the safety of the hydrogel when used as an injectable adhesive.

**Fig. 7:**
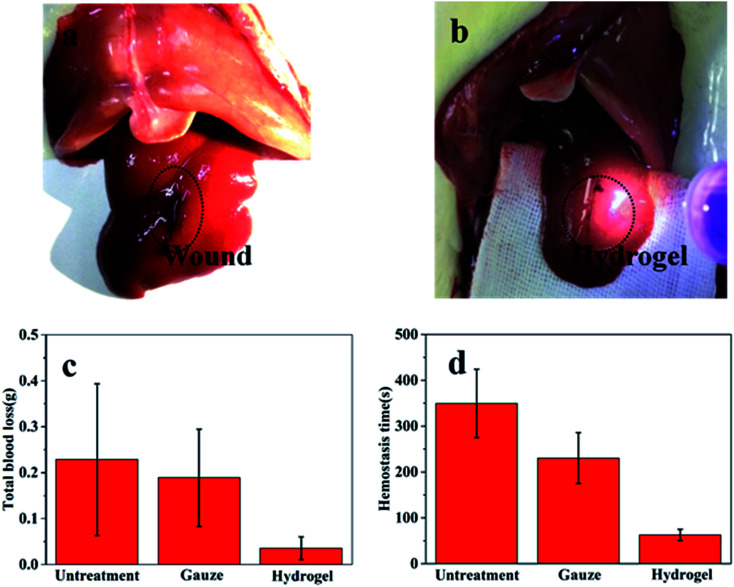
Wound hemostasis test in a rat liver injury model. (a) Bleeding wound untreated; (b) wound treated by the hydrogel; (c) total blood loss until bleeding stopped completely; (d) hemostasis time. The hydrogel is CMC-GEL/OSA containing 5% CMC, 20% GEL, and 15% OSA.

The application of the gel as hemostatic material was evaluated in rat hemorrhaging liver model. The photographs of bleeding wound untreated and treated with the CMC-GEL/OSA hydrogel (5% CMC, 20% GEL, and 15% OSA) were shown in Fig. 7a and b respectively. It could be seen that the hydrogel was adhered onto the liver and the bleeding was effectively arrested. The amount of blood loss and the hemostasis time were measured after applying hydrogel, gauze pad, or no treatment (Fig. 7c and d). The total blood loss was 0.228 g and 0.036 g from the untreated and CMC-GEL/OSA hydrogel treated liver, the blood loss was reduced by 84.2%. The hemostasis time of the hydrogel treated wound was decreased to 62 s from 349 s of the untreated wound, shortened by 82.2%. Compared with gauze pressing, the hydrogel had absolute superiority with less blood loss and shorter hemostasis time. The hemostatic property of CMC-GEL/OSA hydrogel is due to the synergistic effect of the good adhesiveness of the hydrogels and the hemostatic property of carboxymethyl chitosan and gelatin. The results demonstrate that the CMC-GEL/OSA hydrogels have effective hemostasis ability and potential for further application in clinical operation.

## 4. Conclusions

An attractive new adhesive hydrogel consisting of carboxymethyl chitosan, gelatin and oxidized alginate was developed, taking the privilege of the mild condition offered by Schiff base reaction. The hydrogel could form rapidly *in situ* within 30 s and demonstrated good adhesion strength. Good antibacterial property could be obtained *via* the load and release of levofloxacin. When applied to a rat liver wound, the hydrogel showed excellent hemostatic efficacy with 84.2% decrease of bleeding time and 82.2% reduction of blood loss contrast to blank control. These kinds of *in situ* forming hydrogels are expected to be applied for novel tissue adhesives and hemostatic materials and also hold high potential for drug delivery and tissue regeneration.

## Conflicts of interest

There are no conflicts to declare.

## Supplementary Material
